# Application of Mass Spectrometry Technology to Early Diagnosis of Invasive Fungal Infections

**DOI:** 10.1128/JCM.01655-16

**Published:** 2016-10-24

**Authors:** Alexandre Mery, Boualem Sendid, Nadine François, Marjorie Cornu, Julien Poissy, Yann Guerardel, Daniel Poulain

**Affiliations:** a SATT Nord-de-France, Lille, France; b Université Lille, CNRS, UMR 8576-UGSF-Unité de Glycobiologie Structurale et Fonctionnelle, Lille, France; c CHU Lille, Service de Parasitologie-Mycologie, Lille, France; d Université Lille, U995-LIRIC-Lille Inflammation Research International Centre, Lille, France; e INSERM, U995-Team 2, Lille, France; f CHU Lille, Pôle de Réanimation, Lille, France; g CHU Lille, Délégation à la Recherche Clinique et à l'Innovation, Lille, France; University of Iowa College of Medicine

## Abstract

We recently developed a mass spectrometry (MS) procedure based on the detection of a serum disaccharide (MS-DS) in patients with invasive candidiasis (IC). Here, we compare the performance of MS-DS for the diagnosis of IC, invasive aspergillosis (IA), and mucormycosis (MM) with those of commercially available antigen detection tests. This retrospective study included 48 patients (23 IC patients [74 serum samples], 15 IA patients [40 serum samples], and 10 MM patients [15 serum samples]) and 49 appropriate controls (102 serum samples). MS-DS, mannan (Mnn), galactomannan (GM), and (1,3)-β-d-glucan (BDG) were detected by matrix-assisted laser desorption ionization–time of flight (MALDI-TOF) MS, Platelia, and Fungitell assays, respectively. For IC, the sensitivity and specificity of the MS-DS index, BDG detection, and Mnn detection were 62% and 84%, 82% and 60%, and 33% and 94% per serum sample and 83% and 69%, 96% and 31%, and 39% and 86% per patient, respectively. For IA, the corresponding values in comparison to BDG and GM detection were 83% and 81%, 62% and 95%, and 62% and 100% per serum sample and 93% and 76%, 87% and 90%, and 93% and 100% per patient, respectively. Nine of the 10 MM patients had a positive MS-DS result. MS-DS gave an early diagnosis in IC (73% positivity before blood culture), IA (positive before GM detection in six patients), and MM (positivity mainly preceded the date of diagnosis) patients. For IC, persisting MS-DS was associated with a poor prognosis. The different biomarkers were rarely detected simultaneously, suggesting different kinetics of release and clearance. For IA, MS-DS provided better complementation to GM monitoring than BDG monitoring. MS-DS detects panfungal molecules circulating during invasive fungal infections. The performance of MS-DS compared favorably with those of biological tests currently recommended for monitoring at-risk patients. Further validation of this test in multicenter studies is required.

## INTRODUCTION

Invasive candidiasis (IC) and invasive aspergillosis (IA) are major life-threatening nosocomial invasive fungal infections (IFIs) (1–3). Although less prevalent, mucormycosis (MM) is an emerging problem. Progress in antifungal therapy has not significantly reduced the high rates of morbidity and mortality associated with IFIs, particularly in intensive care units (ICUs) and oncohematology units (4–6), due to difficulties in obtaining an early diagnosis, an important condition for a favorable outcome (7). Difficulties in the biological detection of IFIs are related to the low yield of culture-based methods (8); blood cultures are positive in only ∼50% of episodes of IC and in anecdotic cases of IA. To fill this gap, methods have been developed for the detection of fungal molecules in sera from patients (9–11). These methods include the detection of fungal DNA in body fluids and tissues, for which no consensual recommendations have been produced due to the lack of standardization. In contrast, there is extensive literature on the diagnostic value of fungal polysaccharide detection, including (1,3)-β-d-glucan (BDG) (12), present in Candida and Aspergillus cell walls, and mannan (Mnn) or galactomannan (GM), found in Candida and Aspergillus, respectively. Each of these assays, which present different compromises between sensitivity and specificity, are currently widely used, although there is a lack of consensus about therapeutic decisions based on the results of these tests in the complex setting of IC and IA (13–15). For MM, no serological test is currently of diagnostic help.

In a previous report, we described the presence of a specific *m/z* 365 matrix-assisted laser desorption ionization (MALDI) mass spectrometry (MS) signal in sera of IC patients. The fungal origin of this signal, identified as dihexasaccharide (DS), was confirmed in an experimental model of IC (16). In the present study, we evaluated the clinical usefulness of this new biomarker in well-characterized cohorts of patients with IC, IA, and MM, with reference to appropriate hospitalized controls and in comparison with BDG, Mnn, and GM detection tests.

(This work was presented as an oral communication at the 26th European Congress of Clinical Microbiology and Infectious Diseases, Amsterdam, Netherlands, 9 to 12 April 2016.)

## MATERIALS AND METHODS

### Study population. (i) Patients with IFI.

Patients were selected from the database of Lille University Hospital according to the following criteria: (i) classification as having proven or probable IFI according to European Organization for the Research and Treatment of Cancer/Mycoses Study Group (EORTC/MSG) criteria (17) and (ii) availability of sera drawn around the time when clinical/mycological/imaging evidence of IFI was obtained. The clinical and biological characteristics of the patients with IC, IA, and MM are shown in Tables 1, 2, and 3, respectively.

**TABLE 1:** Clinical and biological characteristics of patients with IC^*a*^

Patient	Sex	Patient age (yr)	Hospital ward	Underlying condition(s)	No. of serum samples (no. of serum samples before BC)	Serum sampling points (time to BC) (days)	Candida species isolated from BC	BDG concn (pg/ml) (min–max)	Mnn concn (pg/ml) (min–max)	MS-DS index (%) (min–max)	Outcome	
											Death within 1 mo	Hospital death
1	M	58	ICU	Perineal cellulitis and digestive cancer	3 (1)	−5, 1, 6	C. albicans	315–912	236–2,039	800–1,000		Yes
2	M	37	ICU	Post-heart-graft care	3 (2)	−3, 0, 7	C. albicans	71–113	0	350–2,000		
3	M	49	ICU	Digestive cancer, septic shock postchemotherapy, type 2 diabetes	3 (1)	−3, 4, 18	C. albicans	1,088–1,704	373–1,859	500–1,000		Yes
3					3 (1)	−4, 3, 10	C. albicans	264–341	363–435	500–650		
4	M	70	Hematology	Lymphoma, DRESS syndrome	4 (2)	−2, 0, 3, 56	C. albicans	170–364	0–2,500	77–700		
5	F	51	ICU	Septic shock with Staphylococcus aureus	3 (1)	−2, 5, 12	C. albicans	816–1,712	0	220–700		Yes
6	F	56	ICU	Autoimmune hepatitis, immunosuppressive therapy, corticosteroids, liver fibrosis	3 (2)	−3, −2, 3	C. dubliniensis	0–1,488	0	115–800	Yes	Yes
7	M	66	Surgery	Pulmonary cancer, scleroderma, postsurgery care	3 (2)	−2, 0, 12	C. albicans	82–311	0	141–800		Yes
8	F	60	ICU	Ingestion of caustic substances, gastrectomy	3 (3)	−8, −7, 0	C. albicans	536–2,832	0–614	220–700	Yes	Yes
9	M	66	ICU	Pituitary macroadenoma, chronic respiratory failure, septic shock, type 2 diabetes	4 (2)	−1, 0, 7, 35	C. albicans	1,872–3,992	0–2,500	270–500		Yes
10	M	60	ICU	Post-heart-graft care, immunosuppressive therapy, corticosteroids	3 (1)	−1, 4, 14	C. albicans	0–323	0	200–1,000		
11	M	41	ICU	Burns, alcoholism	4 (2)	−3, 0, 4, 36	C. albicans	27–2,720	0	62–300		
12	F	59	ICU	Mesenteric ischemia, cardiopulmonary arrest	3 (1)	0, 6, 12	C. glabrata	324–3,904	0–6	230–1,000	Yes	Yes
13	F	81	Surgery	Mesenteric vein thrombosis, type 2 diabetes	3 (1)	−25, 5, 12	C. glabrata	63–101	0–77	400–800		Yes
14	F	47	Hematology	AML, HSC allograft, immunosuppressive therapy, nucleoside analogue, COPD	3 (1)	−5, 2, 9	C. glabrata	0–32	0	143–350		
15	F	57	Hematology	AML, HSC allograft, hepatosplenic candidiasis	3 (2)	−6, 0, 3	C. glabrata	329–468	0	192–280		
16	F	52	Burns	Hydrocephalus of undetermined origin, burns	2 (1)	−18, 3	C. parapsilosis	139–720	0	400–700		
17	M	69	ICU	Chronic cardiac failure, post-heart-surgery care, type 2 diabetes	3 (1)	0, 4, 11	C. parapsilosis	30–89	0	230–270		Yes
18	M	69	ICU	Postsurgery care for pacemaker infection, septic shock	3 (1)	0, 2, 9	C. parapsilosis	760–1,464	0	450–1,000		Yes
19	F	34	Hematology	AML chemotherapy, nucleoside analogue	3 (2)	−6, 1, 6	C. tropicalis	11–108	0	300–450		
20	M	61	Gastroenterology	Liver fibrosis, hepatorenal syndrome	3 (2)	−4, 0, 7	C. tropicalis	60–446	0–507	230–1,500	Yes	Yes
21	M	63	ICU	Post-heart-graft care	3 (2)	−4, −1, 3	C. tropicalis	185–2,096	0–76	380–450	Yes	Yes
22	M	66	ICU	ENT cancer, COPD, septic shock	3 (2)	−12, −5, 2	C. tropicalis	255–333	800–1,065	68–240		
23	F	59	ICU	CLL, HSC allograft, digestive GVHD, immunosuppressive therapy, corticosteroids, chronic hepatitis B	3 (2)	−4, −1, 1	C. krusei	113–261	0	400–500	Yes	Yes

a M, male; F, female; BC, blood culture; BDG, (1,3)-beta-d-glucan; Mnn, mannan; ICU, intensive care unit; AML, acute myeloid leukemia; HSC, hematopoietic stem cell; COPD, chronic obstructive pulmonary disease; ENT, ear, nose, and throat; CLL, chronic lymphocytic leukemia; GVHD, graft-versus-host disease.

**TABLE 2:** Clinical and biological characteristics of patients with IA^*a*^

Patient or serum sample	Hospital ward	Underlying condition(s)	Length of neutropenia (<500 neutrophils/mm^3^) (days)	TDM finding(s)	Patient age (yr)	Sex	Time between collection of serum samples (days)	No. of serum samples positive for GM (consecutive)	BAL fluid culture result	BDG concn (pg/ml)	GM ratio	MS-DS index (%)	IA classification	Outcome (death)	Prophylaxis
P1	Hematology	CLL, HSC allograft, Richter syndrome, corticosteroids, MAb	<10	NA	54	F		2 (2)	Negative	293	0.7	700	NA	Yes	No
P2-1	Hematology	Hodgkin's lymphoma, HSC allograft, nucleoside analogue	>10	Dense lesions without halo sign, regression after 3 wk	23	F	0	12 (12)	Negative	108	0.1	500	Probable		Yes
P2-2							12			147	0.4	1,000			
P2-3							18			62	1.5	300			
P2-4							39			23	0.7	82			
P3-1	Hematology	Lymphoma, HSC allograft, corticosteroids, nucleoside analogue	0	Reversal of ground-glass lesions, micronodules under treatment, budding trees	63	M	0	11 (8)	NA	73	0.3	370	Probable	Yes	Yes
P3-2							9			89	0.73	588			
P3-3							18			104	2.5	850			
P4-1	Hematology	Myelofibrosis on essential thrombocythemia, HSC allograft, nucleoside analogue, cyclosporine	>10	Reversal of dense lesions and ground glass upon antifungal therapy	63	M	0	15 (12)	Negative	83	1.3	210	Probable		No
P4-2							49			306	0.7	1,500			
P4-3							106			324	0.3	3,000			
P5-1	Digestive surgery	Digestive postsurgical complications	0	Stable nodules	65	M	0	5 (3)	NA	165	0.07	526	NA		No
P5-2							53			166	2.6	650			
P5-3							56			65	0.4	556			
P6	ICU	Thymoma, myeloma, septic shock, chemotherapy	>10	Ground glass, atelectasis	79	M		1 (1)	Aspergillus fumigatus	>500	6	1,500	Probable	Yes	No
P7	ICU	Lymphoma, HSC allograft, MAb, nucleoside analogue	>10	Micronodules, atelectasis, excavation	59	M		3 (3)	A. fumigatus	342	0.8	1,000	Probable		Yes
P8	ICU	Heart valve replacement postsurgical care, cardiogenic shock	0	NA	70	M		5 (5)	NA	143	5.2	550	NA	Yes	No
P9-1	Oxygen therapy	Necrotizing fasciitis of legs, septic shock	0	Dense lesions and ground glass (ARDS corresponding)	43	F	0	0	A. fumigatus	222	0.25	54	Probable	Yes	No
P9-2							11			300	0.1	54			
P10-1	Cardiovascular surgery	Cardiac transplant, corticosteroids	0	Dense lesions with halo	38	M	0	7 (5)	A. fumigatus	500	0.3	2,000	Proven		No
P10-2							4			500	0.6	1,000			
P10-3							21			500	5	1,500			
P11-1	Hematology	Myelodysplasia, Hodgkin's lymphoma, HSC allograft, corticosteroids, immunosuppressive therapy	>10	Dense lesion	48	F	0	6 (3)	NA	0	0.1	400	Probable		NA
P11-2							7			12	0.9	182			
P11-3							14			478	2	400			
P12-1	Hematology	AML, HSC allograft	>10	Ground-glass, dense lesions with halo	64	F	0	16 (16)	Negative	0	0.05	370	Probable	Yes	Yes
P12-2							14			125	0.21	500			
P12-3							20			15	2.71	833			
P12-4							24			177	1.47	588			
P12-5							42			120	0.75	5,000			
P12-6							56			95	1.21	1,000			
P13-1	Hematology	AML, nucleoside analogue	>10	Ground glass	69	M	0	2 (2)	NA	10	0.03	200	Possible	Yes	NA
P13-2							13			0	0.32	526			
P13-3							21			0	0.55	714			
P13-4							23			66	1.13	500			
P14-1	Hematology	Burkitt's lymphoma, HSC allograft	>10	Dense lesion	23	M	0	9 (5)	NA	0	0.07	500	Probable	Yes	NA
P14-2							15			0	2.4	500			
P14-3							50			0	1.26	500			
P15-1	Hematology	AML, HSC allograft, corticosteroids, cyclosporine	0	Ground glass	56	M	0	2 (2)	NA	265	0.5	833	Possible	Yes	NA
P15-2							5			226	2.1	1,000			

a M, male; F, female; TDM, tomodensitometry; BAL, bronchoalveolar lavage; BDG, (1,3)-beta-d-glucan; GM, galactomannan; ICU, intensive care unit; CLL, chronic lymphocytic leukemia; HSC, hematopoietic stem cell; MAb, monoclonal antibody; AML, acute myeloid leukemia; NA, not available.

**TABLE 3:** Clinical and biological characteristics of patients with mucormycosis^*c*^

Patient	Hospital ward	Patient age (yr)	Sex	Underlying condition(s)	Clinical type/site of mucormycosis (sample for diagnosis)	TDM finding(s)	Microorganism	EORTC/MSG group classification	No. of days from diagnosis (serum sample)	BDG concn (pg/ml)	GM ratio	MS-DS index (%)	Outcome
M1	Hematology	61	F	HSC allograft after myelodysplasia	Pulmonary (lung biopsy specimen)	Dense lesion, right upper lobe	Rhizopus microsporus^*b*^	Proven	−4 (L1-M1)	0	0.07	132	Death
									+12 (L2-M1)			**2,000**	
M2	Transplant	66	M	Liver fibrosis, corticosteroids, diabetes, liver graft	Rhinosinus (sinus, face, nose)		Rhizopus pusillus^*b*^	Proven	−1 (L3-M2)			**588**	Death
M3	Burns	66	F	Burns, CML	Skin (skin swab)		Lichtheimia ramosa		−2 (L4-M3)	31		132	Alive
									+5 (L5-M3)	18		159	
M4	Burns	42	M	Burns	Skin (biopsy specimen)		Lichtheimia corymbifera	Proven	−1 (L6-M4)	**146**		182	Alive
									+6 (L7-M4)	**203**		**2,000**	
M5	Hematology	45	F	HSC allograft after AML, GVHD stage IV	Postoperative abscess of abdominal wall (biopsy specimen)		Rhizopus arrhizus^*b*^	Proven	−9 (L8-M5)	42	0.15	**300**	Death
									+6 (L9-M5)	46		**2,000**	
M6	Burns	42	M	Burns	Skin (biopsy specimen)		Lichtheimia corymbifera	Proven	+24 (L10-M6)	21		**1,429**	Alive
M7	Hematology	83	F	Mantle cell lymphoma, rituximab, diabetes	Lung (BAL fluid)	Dense lesion, right middle lobe	Rhizopus microsporus	Probable	−6 (L11-M7)	18	0.06	**455**	Death
									0 (L12-M7)	0	0.04	**400**	
M8	ICU	76	M	Trauma	Skin (biopsy specimen)		Mucor circinelloides	Proven	+4 (L13-M8)	39		**3,333**	Alive
M9	ICU	60	M	Trauma	Skin (biopsy specimen)		Mucor circinelloides	Proven	+5 (L14-M9)	**106**	0.06	**3,333**	Alive
M10	Hematology	3	F	ALL B (induction)	Disseminated, brain, eye, lung, kidney, calf (vitreous humor, muscle biopsy specimen)		Lichtheimia sp.^*a*^	Proven	−1 (L15-M10)	18	0.05	**333**	Alive

a Microorganisms were obtained in culture except for the last one, where the diagnosis was made by quantitative PCR.

b Samples positive by histology.

c Boldface type indicates a positive value. ICU, intensive care unit; M, male; F, female; HSC, hematopoietic stem cell; CML, chronic myeloid leukemia; AML, acute myeloid leukemia; GVHD, graft-versus-host disease; ALL, acute lymphocytic leukemia; BAL, bronchoalveolar lavage; TDM, tomodensitometry; BDG, (1,3)-beta-d-glucan; GM, galactomannan; DS, serum disaccharide.

### (ii) Control subjects.

Control subjects consisted of hospitalized patients in ICUs and hematology wards who were considered appropriate controls with major risk factors for IFIs. In the ICU, we selected 29 control patients for IC who had previously been enrolled in a prospective study and for whom 82 serum samples were drawn sequentially in parallel with the determination of the colonization index (see Table S1 in the supplemental material). In oncohematology wards, controls for IA consisted of 20 patients for whom regular monitoring of GM was performed (1 serum sample per patient) (see Table S2 in the supplemental material). Both groups corresponded to suitable controls for MM.

### Measurement of mannan and glucan polysaccharides/oligosaccharides.

The BDG concentration was measured by using the Fungitell kit (Associates of Cape Cod Inc., Falmouth, MA, USA) according to the manufacturer's instructions. The recommended cutoff of 80 pg/ml was used to determine clinical relevance. Measurement of serum Mnn levels was performed by using the Platelia Candida Ag^+^ test (Bio-Rad, Marnes la Coquette, France) according to the manufacturer's instructions. The recommended cutoff of 62.5 pg/ml was used to determine clinical relevance.

For both tests, serum samples with positive results of >500 pg/ml were diluted and retested.

### Detection of DS by MS.

The procedure used for the detection of DS was described previously (16). The same procedure was applied here, using a 4800 MALDI-TOF/TOF analyzer (Applied Biosystems/MDS Sciex) at a fixed laser intensity and 1,000 accumulated shots/spectrum within an *m/z* 300 to 800 range. The reproducibility and repeatability of the test were assessed in double-blind studies involving at least two different laboratories; the coefficients of variation were <10% and <5%, respectively. The influence of different mass spectrometers and modes of acquisition (reflectron and/or linear) was determined by using Sciex Voyager DE, Sciex 4700, Bruker Ultraflex Shimadzu Vitek-MS, and Thermo MALDI-LTQ-Orbitrap machines; all of them gave similar results.

### Ethics statement.

All sera used in this study were obtained from patients monitored at Lille University Hospital. When no results were available from routine tests, BDG and mannan levels were determined retrospectively from residual frozen samples. No additional sampling was necessary. As sera were taken from a registered biological collection, patient consent was not required according to French law. Agreement for the establishment of a biological collection of IFI samples was obtained from the French Ministry of Education and Research under reference DC-2008-642. Institutional review board approval was given by the Comité de Protection des Personnes Nord-Ouest IV, the ethical committee of our institution.

### Statistical analysis.

The Mann-Whitney two-tailed test was used to compare the distributions of biomarkers in the different groups, and the nonparametric correlation test (Spearman's rank test) was used to analyze the correlation between them. GraphPad Prism 6 was used to generate receiver operating characteristic (ROC) curves and derive cutoffs and graphs.

A *P* value of <0.05 was considered to be statistically significant.

## RESULTS

### Signal of interest and study design.

The principle of the MALDI-time of flight (TOF) MS procedure as well as the methodology used for the serological diagnosis of IC were described previously (16). This procedure reveals an *m/z* 365 signal, corresponding to a hexadisaccharide (DS), specifically associated with human and experimental IC. Quantification of the signal was performed by establishing the “MS-DS index,” defined as the ratio of *m/z* 365 over *m/z* 361 matrix signal intensities (percent *m/z* 365 versus *m/z* 361). The same intense signal was observed during IA and MM (data not shown). Investigations involving the MS-DS index as a tool for diagnosing IC were therefore carried out. In such a complex setting, the 100% specificity achieved by referring to healthy blood donors as controls is not relevant; thus, in this pilot study, special care was taken to include the most appropriate control groups, which consisted of hospitalized patients at high risk of IFI.

### MS-DS and diagnosis of IC.

The clinical and biological characteristics of the patients with IC are shown in Table 1 (those of the appropriate IC controls are shown in Table S1 in the supplemental material). Both groups were recruited at the same ICU wards. Their clinical characteristics in terms of risk factors were recorded as described previously (18); the two groups did not exhibit any significant differences in terms of major risk factors (acute physiology and chronic health evaluation [APACHE] score, surgery, neutropenia, antibiotherapy, bacteremia, and central venous catheter, etc.), except for mechanical ventilation (100% for the IC group versus 65.2% for the control group; *P* = 0.008) and its duration as well as antifungal therapy (78.3% versus 27.6%; *P* < 0.001). Regarding the mycology results, the median delay between hospital admission and positive blood culture results was 20 days (interquartile range 1 [IQR1], 12.3 days; IQR3, 27.5 days). Candidemia was due to Candida albicans (46%), C. glabrata (17%), C. tropicalis (17%), C. parapsilosis (12%), and miscellaneous species (8%). In the control group, colonization was due to C. albicans (69%), C. parapsilosis (24%), C. tropicalis (14%), C. glabrata (10%), and miscellaneous species (14%). Therefore, the relative prevalence of the different Candida species found in the control group was similar to that in the IC group, with a predominance of C. albicans, followed by C. tropicalis, C. glabrata, and C. parapsilosis.

**FIG 1:**
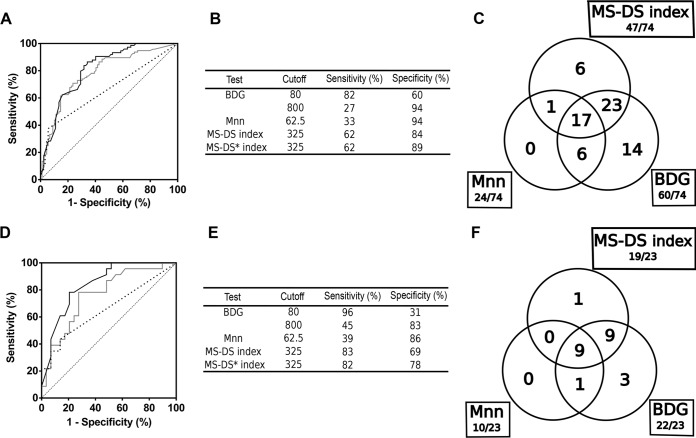
Comparison of BDG detection, Mnn detection, and the MS-DS index. (A to C) Analysis per serum sample. (A) ROC curves based on a comparison between the IC group and the corresponding control group. Dotted line, Mnn detection; gray line, BDG detection; black line, MS-DS index. (B) Cutoffs established by ROC curves and corresponding sensitivity/specificity values. (C) Venn diagrams. (D to F) Analysis per patient. *, control sera exhibiting high levels of BDG (>800 pg/ml) and/or high levels of Mnn (>62.5 pg/ml) were excluded.

Assessment of the diagnostic value of BDG detection, Mnn detection, and the MS-DS index was performed by comparing the IC and control groups (Fig. 1). Analyses were initially made per serum sample (Fig. 1A to C). ROC curves (Fig. 1A) showed a higher area under the curve (AUC) for the MS-DS index (0.81) than for BDG and Mnn detection (0.78 and 0.66, respectively). Using ROC curves, the best sensitivity/specificity compromise (Fig. 1B) for the MS-DS index (62%/84%) was established for a cutoff at 325%. For the same population, this sensitivity was lower than that for BDG detection (82%) but higher than that for Mnn detection (33%), while the converse was found for specificity (60% and 94%, respectively). The Venn diagram showing overlapping positive values revealed that the MS-DS index was positive alone for only 6/74 serum samples and that all other MS-DS index-positive test results were associated with positivity of either Mnn detection, BDG detection, or both. Analysis by patient (Fig. 1D to F) by applying the 325% cutoff for the MS-DS index confirmed a larger AUC for this test (0.84) than for BDG detection (0.76) and Mnn detection (0.66), with a sensitivity per patient of 83% but a decreased specificity of 69%; this trend was also observed for BDG and Mnn detection.

The Venn diagram showed that most of the patients (19/23) were positive by at least two tests, while 4 were positive by only a single test (3 for BDG detection and 1 for the MS-DS index). Consideration of the decrease in specificity per patient as well as the Venn diagram constructed by using controls (data not shown) suggested that some of these controls could be infected with Candida species despite their negative blood culture results. Considering that a previous study established that BDG levels of >800 pg/ml and Mnn levels of >125 pg/ml are indicative of IC (18), we reanalyzed the performance of the MS-DS index by excluding the controls accordingly. As shown in Fig. 1B and E, the application of these criteria to the MS-DS index resulted in an increase in specificity (89% per serum sample and 78% per patient).

**FIG 2:**
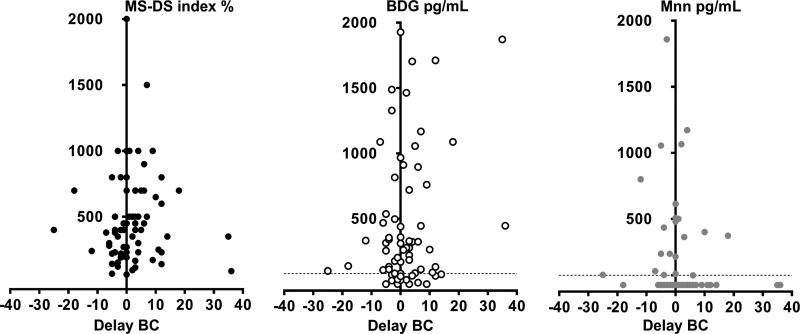
Distribution of MS-DS index, BDG, and Mnn values in sera from IC patients as a function of the date (in days) in relation to the isolation of Candida (day 0). BC, blood culture.

The levels of biomarkers as a function of the date of serum sampling in relation to the date of isolation of Candida species from blood cultures are shown in Fig. 2. All of the biomarkers displayed a Gaussian distribution, with a maximum on day 0, suggesting that the development of candidemia was crucial to their appearance. All tests were positive before blood cultures were performed. Among the 38 serum samples available from 23 patients, 55%, 81%, and 37% were positive by the MS-DS index, BDG detection, and Mnn detection, respectively (corresponding to 17, 20, and 9 patients, respectively). The MS-DS index test was positive up to 25 days, with a median of 3 days, before blood cultures. After blood cultures became positive, there was a trend toward the persistence of BDG detection in contrast to Mnn detection and the MS-DS index. A correlation analysis between MS-DS results and BDG and Mnn results showed that these biomarkers do not circulate at the same time in a given serum sample from a given patient (data not shown). Although the study was retrospective and was based on 3 to 5 available serum samples per patient, information could be obtained about the variation in the MS-DS index compared to BDG and Mnn detection. Representative examples based on a different duration of the survey are shown in Fig. S1 in the supplemental material, illustrating that all biomarkers may appear or disappear abruptly within short periods of time ranging from 1 to 4 days (patients 4 and 6). Conversely, some biomarkers may persist for periods of up to 5 weeks, while others are negative. As these interindividual differences between biomarkers may reveal different kinetics of release and catabolism, we explored the incidence of neutropenia as a characteristic of IC in at-risk patients. No correlation was found between the MS-DS index and polymorphonuclear neutrophil counts (*r*^2^ = 0.1). We also investigated the incidence of colonization on the MS-DS index in control patients for whom the fungal load was known on each day of serum sampling. No correlation was observed between colonization and the MS-DS index (data not shown).

**FIG 3:**
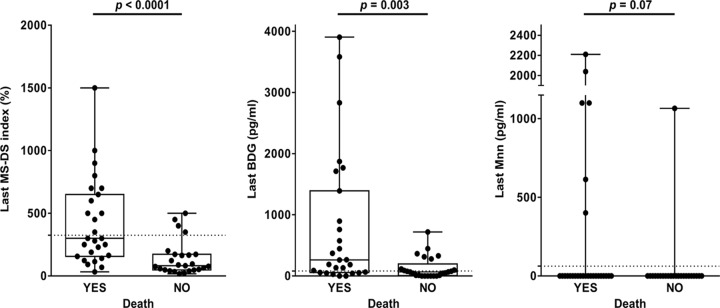
Relationship between outcome and last available value for the MS-DS index, BDG detection, and Mnn detection observed during monitoring of the whole cohort. The hatched line represents the cutoff value for each biomarker.

Analysis of the relationship between biomarker levels and the outcome of IC was also performed. Figure 3 shows the last available value for each biomarker as a function of survival at 1 month. Mortality in IC patients was significantly associated with a high serum MS-DS index and high levels of BDG, with a greater significance for the MS-DS index (*P* < 0.0001 by a Mann-Whitney test).

### MS-DS and diagnosis of IA.

The clinical and biological data for the patients with IA are shown in Table 2, and those of the corresponding controls are shown in Table S2 in the supplemental material. The studied population consisted of 15 patients with IA and 20 controls. Most of the patients had underlying hematological problems. The control group consisted of patients who were hospitalized in oncohematology wards and exposed to the same risk factors as those for IA patients and for whom regular surveillance of galactomannanemia was performed.

**FIG 4:**
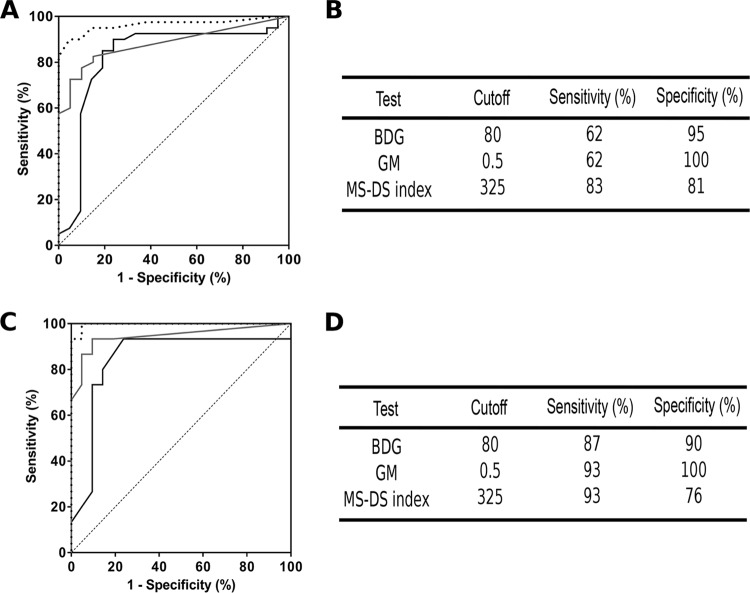
(A) ROC curves per serum sample in IA patients and the corresponding control group. (B) Established cutoffs from ROC curves and sensitivity/specificity values for BDG detection (gray line), GM detection (dotted line), and the MS-DS index (black line) for analysis per serum sample. (C) ROC curves per patient for IA patients and the corresponding control group for GM detection, BDG detection, and the MS-DS index. (D) Established cutoffs from ROC curves and sensitivity/specificity values for BDG detection, GM detection, and the MS-DS index for analysis per patient.

The contribution of the MS-DS index to the diagnosis of IA was evaluated by establishing ROC curves per serum sample in comparison to BDG and GM detection (Fig. 4A and B). Determination of the cutoff showed that the MS-DS index was lower than that for IC (290%). However, as this led to only a slight improvement in sensitivity (85%), the same cutoff as that for IC was used (325%) to maintain homogeneity. When analyzed per patient, the specificity of the MS-DS index decreased slightly to 76%, while the sensitivity increased to 93% (Fig. 4C and D). These values are similar to those for BDG detection but lower than those for GM detection, which was chosen as one of the inclusion criteria.

With regard to the controls, one patient who exhibited a very high MS-DS index (1,500%) was among two controls classified as having “possible IA.” As neither of these two patients were positive for BDG or GM, the MS-DS index in this case might have been an early indicator of IA.

When considering the 40 serum samples collected from patients with IA, 33, 25, and 25 were positive by the MS-DS index, BDG detection, and GM detection, respectively. Analysis of the contribution of each marker to the early diagnosis of IA was performed by using sera taken on the first week of GM monitoring. This analysis showed that 4, 8, and 10 serum samples were positive by GM detection, BDG detection, and the MS-DS index, respectively. Considering the distribution of the biomarkers, no correlation was observed between the MS-DS index and BDG detection (*r* = 0.028) or the MS-DS index and GM detection (*r* = 0.21), indicating that these biomarkers do not circulate at the same time in a given serum sample from a given patient.

This high interindividual variability of biomarker kinetics is summarized in Fig. S2 in the supplemental material, with representative examples of biomarker kinetics during IA episodes. Patient 4 had a decrease in the GM level, whereas his MS-DS index increased constantly during the time of the survey. In contrast, patient 11 had an increase in the GM level, while her MS-DS index remained positive until the end of the survey. However, concordant kinetics could also exist, as for patient 3.

The MS-DS index in IA patients was significantly higher than that observed for IC patients (826% versus 471%).

### MS-DS and diagnosis of MM.

The clinical and biological data for patients with MM are shown in Table 3. These cases were collected in our clinical laboratory from 2015 to 2016. These patients presented the usual characteristics of patients infected with Mucorales in terms of underlying conditions, including immunosuppression, burns, and trauma, as well as the causative agents isolated.

Nine of these patients had a high MS-DS index. Most of the sera were collected retrospectively after the time of diagnosis of MM, and serum samples were available for only five of these patients before or on the day of diagnosis; four of these patients were positive. Although few sequential serum samples were available per patient, two MS-DS conversions were observed. Interestingly, the MS-DS index observed during MM was very high.

## DISCUSSION

IFIs are severe life-threatening diseases and are a serious medical problem in immunocompromised patients. IC and IA account for 73.4% and 13.3% of IFIs, respectively (1). These infections are difficult to diagnose and are often characterized by a fulminant evolution and death (19, 20). Although MM has a lower prevalence (1.6%), this infection, caused by primitive molds slowly evolving in ulceronecrotic lesions, generates peculiar attention from the medical community because its late diagnosis usually results in death or debilitating surgery.

There is extensive literature on IC and IA, and a number of recommendations have been reported for the management of at-risk patients (10, 21). The high economic impact of inappropriate antifungal therapy in this setting has been particularly well documented (22, 23). For IC, a novel culture-independent technology based on DNA detection by T2 magnetic resonance and nanotechnologies has been proposed to detect and identify the causative yeast directly in patient samples (24). Application of this technology considerably reduces the delay in diagnosis in comparison to blood cultures, which is associated with increased hospital mortality and cost. When adopting an institution-wide T2Candida testing strategy instead of a blood culture-based strategy, potential savings of $1,148 per tested patient and a reduction in mortality of 61% have been observed (24). In clinical circumstances of patients with intra-abdominal Candida infection or patients who have received antifungal therapy (for patients with proven or suspected IC in the absence of candidemia), the high analytical sensitivity (1 to 3 CFU/ml) generates an increase in sensitivity of 36% per patient (22). With regard to the delay in diagnosis in relation to blood cultures, T2Candida reduced the mean time to detection and species identification from 129.9 to 4.4 h. To address the problem of a reduction in time to diagnosis in order to initiate appropriate antifungal treatment, many studies have been carried out on the detection of circulating fungal molecules, which may complement blood cultures and discriminate IC patients from controls with similar risk factors without documented IFI. This approach led to the development of serological tests as adjuncts when making therapeutic decisions. In this setting, meta-analyses of clinical studies have led to moderate-level recommendations concerning the use of BDG and Mnn tests and of Mnn and anti-Mnn tests combined, as suggested previously (10, 25, 26). For IA, the use of GM and BDG tests is recommended (17, 26). Based on our previous study identifying a new biomarker (MS-DS) detected by MALDI-TOF MS (16), we carried out a large-scale evaluation of this method for the diagnosis of IC, IA, and MM with appropriate hospital controls.

For the diagnosis of IC per patient, application of a cutoff of 325% was associated with a sensitivity and specificity of 83% and 69%, respectively; the sensitivity was higher than that of Mnn detection (39%), and the specificity was higher than that of BDG detection (31%). When the MS-DS index cutoff was decreased to 260% in order to reach the same sensitivity as that of BDG detection (96%), the specificity of MS-DS (52%) remained higher than that of BDG detection.

These findings suggest that the MS-DS index positively complements Mnn and BDG detection for the diagnosis of IC. All of these tests were positive before blood culture results were available. Among the 38 serum samples available from 23 patients, 55%, 81%, and 37% were positive by the MS-DS index, BDG detection, and Mnn detection, respectively, which corresponded to 17, 20, and 9 patients, respectively, suggesting the usefulness of the MS-DS index as an early diagnostic marker for IC. The glycobiomarkers exhibited different kinetics during the time course of infection and were rarely positive simultaneously. Analysis of this panel of IFI patients and appropriate controls, regrouping patients from oncohematology wards and/or suffering from bacterial infections due to the usual bacterial species encountered in the hospital environment, showed that neither neutropenia nor bacteremia influenced MS-DS levels. The prognostic significance of BDG persistence or an increasing slope during patient screening has been proposed as an indicator for monitoring treatment of IC (27). By using the last available value for each test in monitored patients as a function of survival at 1 month, it was established that the persistence of the MS-DS index was more significantly associated with an unfavorable prognosis than BDG detection.

When the MS-DS index was evaluated for the diagnosis of IA in comparison to BDG and GM assays, the sensitivities and specificities per patient were 93% and 76%, 87% and 90%, and 93% and 100% for the MS-DS index, BDG detection, and GM detection, respectively. This is in agreement with the best performance reported so far with the BDG and GM tests, although it must be stated that in the present study, the 100% sensitivity of GM detection is related to the choice of GM positivity as an inclusion criterion. In terms of their contribution to the early diagnosis of IA, BDG detection has been shown to give a better performance than GM detection in pediatric and adult neutropenic patients (28, 29). In the present study, the MS-DS index provided earlier positive results than the other tests on the first serum samples available. During the time course of the disease, these biomarkers do not circulate at the same time. GM is a well-recognized surrogate biomarker (26) of IA, and the MS-DS index complemented GM detection more positively than BDG detection.

With regard to MM, a definite diagnosis is based on the histopathology of biopsy specimens from which cultures are often negative (30) (especially in patients who have received preventive or empirical antifungal therapy). Some progress has recently been made in the diagnosis of MM by real-time PCR in specialized centers (31–36), but none of the serological tests currently on the market are efficient for the diagnosis of MM. Our study clearly demonstrates that MS-DS could fill this gap as a new serological marker for MM, becoming positive before the establishment of a clinical diagnosis. Altogether, these results suggest a panfungal nature of this new biomarker, and studies are now in progress to investigate its synthesis and release by fungal cells.

In conclusion, these findings suggest that the MS-DS index is a novel physicochemical diagnostic test for the diagnosis of major IFIs. It is cheap to perform and is easily implementable in the majority of clinical mycology laboratories equipped with a routine MALDI-TOF mass spectrometer. Studies are in progress to validate the robustness of this marker in patient cohorts recruited in different European centers.

## Supplementary Material

Supplemental material
