# Supplementary material for: Application of Mass Spectrometry Technology to Early Diagnosis of Invasive Fungal Infections
Source: J Clin Microbiol. 2016 Oct 24;54(11):2786–97. doi: 10.1128/JCM.01655-16 (PMC5078558; doi:10.1128/JCM.01655-16)
Supplement: Supplemental material [file supp_54_11_2786__index.html]

Supplemental material 

# Application of Mass Spectrometry Technology to Early Diagnosis of Invasive Fungal Infections

## Supplemental material

- Supplemental file 1 -

  Tables S1 (Clinical and biological characteristics of hospitalized control group for invasive candidiasis) and S2 (Clinical and biological characteristics of hospitalized control group for invasive aspergillosis) and Fig. S1 and S2 (Kinetics of serum biomarkers)

  PDF, 464K
