# Supplementary material for: Application of Mass Spectrometry Technology to Early Diagnosis of Invasive Fungal Infections
Source: J Clin Microbiol. 2016 Oct 24;54(11):2786–97. doi: 10.1128/JCM.01655-16 (PMC5078558; doi:10.1128/JCM.01655-16)
Supplement: Supplemental material [file JCM.01655-16_zjm999095207so1.pdf]

Supplemental data.

Table S1. Clinical and biological characteristics of hospitalized (ICU) control group for invasive candidiasis (IC).

Patient ID	Sex	Age	Hospital ward	Underlying condition	No. of sera	*Candida* species	BDG (pg/mL) min-max	Mnn (pg/mL) min-max	MS-DS index (%) min-max	Probability of IC based on biomarkers	Probability of IC based on clinical features*	Probability of IC based on clinical and biological features	Outcome	
													Death within 1 month	Hospital death
24	M	60	ICU	COPD, pulmonary embolism	3	*C. tropicalis*<br>*C. albicans*	0-134	0	50-280					Yes
25	M	72	ICU	Chronic respiratory/heart failure	3	*C. albicans*	12-1392	0	139-350	Yes				Yes
26	M	60	ICU	COPD, *Streptococcus pneumoniae* pneumonia	2	*C. albicans*<br>*C. glabrata*	0-12	0	52-65					
27	M	65	ICU	Mesothelioma	3	*C. parapsilosis*<br>*C. kefyr*	56-101	0	60-164		NA			Yes
28	M	75	ICU	Septic shock, pneumonia, ulcerative colitis	3	*C. albicans*	20-89	0	200-300		Possible			
29	M	72	ICU	Cervical haematoma due to anticoagulant	3		38-120	0	40-400					
30	M	67	ICU	Septic shock, pneumonia, alcoholism	3	*C. parapsilosis*	319-936	0-200	156-550	Yes			Yes	Yes
31	M	62	ICU	COPD, *S. pneumoniae* pneumonia, septic shock	3	*C. albicans*<br>*C. glabrata*<br>*C. tropicalis*	46-215	0	33-350					Yes
32	M	76	ICU	Crush trauma	2		0-9	0	10-47					
33	M	69	ICU	Pneumonia, alcoholism, liver failure	3	*C. albicans*	49-120	0	122-137					

34	M	70	ICU	Lung cancer, COPD, pneumonectomy	3	*C. glabrata*<br>*C. tropicalis*	52-469	0	300-700		Possible			Yes
35	F	64	ICU	Lymphoma, pneumonia	2		11-191	0	172-250		Possible			
36	M	46	ICU	Stomach cancer, septic shock, pneumonia	3	*C. albicans*	25-3584	0	97-700	Yes	Probable	Probable	Yes	Yes
37	F	79	ICU	Allergic asthma, corticosteroids	2	*C. albicans*<br>*C. parapsilosis*	38-77	0	49-280					
38	M	71	ICU	COPD, Parkinson’s disease	3	*C. albicans*	0-87	0	22-300		Possible			Yes
39	M	62	ICU	Burkitt’s ALL, tumour lysis syndrome, cardiopulmonary arrest	3	*C. albicans*	97-188	0	189-1000					Yes
40	M	67	ICU	Septic shock, *S. pneumoniae* pneumonia	3	*C. parapsilosis*	0-65	0	38-164					
41	M	79	ICU	Septic shock, pneumonia	3	*C. albicans*<br>*C. parapsilosis*	17-107	0	83-123					Yes
42	M	88	ICU	COPD, pneumonia	2	*C. albicans*	0-86	0	75-88		NA			
43	M	58	ICU	Septic shock, pneumonia, alcoholism	3	*C. albicans*	0-100	0	62-500				Yes	Yes
44	M	51	ICU	Pneumonia	3	*C. albicans*	0-28	0	22-135					
45	M	43	ICU	Pneumonia, alcoholism, cirrhosis, HCV	3	*C. albicans*	32-66	0	58-130					
46	M	79	ICU	Lymphoma, pneumonia	3	*C. albicans*<br>*C. parapsilosis*	187-1768	0-1100	125-280	Yes	Probable	Probable		Yes
47	F	68	ICU	Septic shock, subcutaneous abdominal infection, corticosteroids	3	*C. kefyr*<br>*C. lusitaniae*	196-1176	0-400	39-1000	Yes				
48	M	61	ICU	Tongue cancer, cervical cellulitis, alcoholism	3	*C. albicans*<br>*C. parapsilosis*	0-62	0	34-53					
49	M	29	ICU	Bronchial spasm post-dental surgery	3	*C. albicans*<br>*C. tropicalis*	31-336	0	82-150		Probable			

50	M	55	ICU	Septic shock, *S. pneumoniae* pneumonia, alcoholism	3	*S. cerevisiae*	152-370	0-1100	69-200	Yes			Yes	Yes
51	M	41	ICU	Attempted suicide, rhabdomyolysis	3	*C. albicans*	0-42	0	43-96					
52	F	47	ICU	Pancreatitis, alcoholism	3	*C. albicans*	0-40	0	17-53					

M, male; F, female; BDG, (1,3)-beta-D-glucan; Mnn, mannan; ICU, intensive care unit; ALL, acute lymphocytic leukaemia; HCV, hepatitis C virus; COPD, chronic obstructive pulmonary disease; NA, not available.

* Control patients hospitalized in ICU were secondarily classified, concerning the probability of IC, according to criteria proposed by Mohr et al. in *Prospective survey of (1,3)-beta-D-glucan and its relationship to invasive candidiasis in the surgical intensive care unit setting*; J Clin Microbiol.; 2011.

Table S2. Clinical and biological characteristics of hospitalized (haematology) control group for invasive aspergillosis (IA).

Patient-serum ID	Underlying conditions	Length of neutropenia	TDM	Age	Sex	No. of sera with positive GM	BAL culture result	BDG (pg/ml)	GM ratio	MS-DS index (%)	IA	Outcome (death)	Prophylaxis
C1	Bone marrow aplasia, HSC allograft	>10 days, <500/mm^3	NA	19	M	0	NA	0	0.05	135	No		Yes
C2	AML, HSC allograft	>10 days, <500/mm^3	NA	53	M	0	NA	0	0.06	96	No		Yes
C3	MDS, HSC allograft, nucleoside analogue, immunosuppressive therapy	>10 days, <500/mm^3	Negative	61	F	0	NA	0	0.1	500	No		Yes
C4	AML, CMML, HSC allograft, nucleoside analogue, cyclosporin	0	NA	64	M	0	NA	0	0.03	123	No		Yes
C5	AML, HSC allograft	9 days, <500/mm^3	NA	58	M	0	NA	0	0.03	179	No		Yes
C6	AML	0	Micronodules	58	F	0	NA	0	0.05	79	No		No
C7	AML, HSC allograft, cyclosporin	>10 days, <500/mm^3	NA	44	F	0	NA	0	0.03	127	No		Yes
C8	AML	>10 days, <500/mm^3	Negative	66	M	0	NA	0	0.04	167	No		Yes
C9	Hodgkin’s lymphoma, HSC allograft, cyclosporin	>10 days, <500/mm^3	NA	48	M	0	NA	0	0.03	280	No		Yes
C10	ALL, HSC allograft, corticosteroids, immunosuppressive therapy	0	NA	45	M	0	NA	44	0.04	2000	No		Yes

C11	AML, HSC allograft, corticosteroids	0	NA	56	M	0	NA	89	0.08	400	No	Yes	Yes
C12	Myeloma, HSC allograft, immunosuppressive therapy	0	NA	52	M	0	NA	0	0.04	152	No		NA
C13	AML, HSC allograft	>10 days, <500/mm^3	NA	68	M	0	NA	0	0.04	147	No		NA
C14	ALL, HSC allograft, cyclosporin	0	NA	16	M	0	NA	0	0.05	108	No		Yes
C15	AML, HSC allograft	0	NA	60	M	0	NA	0	0.04	159	No		NA
C16	AML, HSC allograft, nucleoside analogue	0	NA	68	F	0	NA	0	0.04	137	No		Yes
C17	AML, HSC allograft, cyclosporin, corticosteroids	0	Negative	41	F	0	NA	12	0.07	182	No		No
C19	MDS, HSC allograft, nucleoside analogue, cyclosporin, corticosteroids	0	NA	59	M	0	NA	0	0.04	189	No		Yes
C20	AML, HSC allograft, corticosteroids, cyclosporin	0	Ground glass, dense lesions	45	F	0	NA	0	0.,06	109	Possible	Yes	Yes
C21	AML, HSC allograft, nucleoside analogue	1 day, <500/mm^3	Ground glass	54	M	0	NA	0	0.03	1500	Possible		Yes

M, male; F, female; TDM, tomodensitometry; BAL, bronchoalveolar lavage; BDG, (1,3)-beta-D-glucan; GM, galactomannan; CMML, chronic myelo-monocytic leukaemia; HSC, haematopoietic stem cells; MDS, myelodysplasia; MAb, monoclonal antibody; AML, acute myeloid leukaemia; NA, not available.

Figure S1.

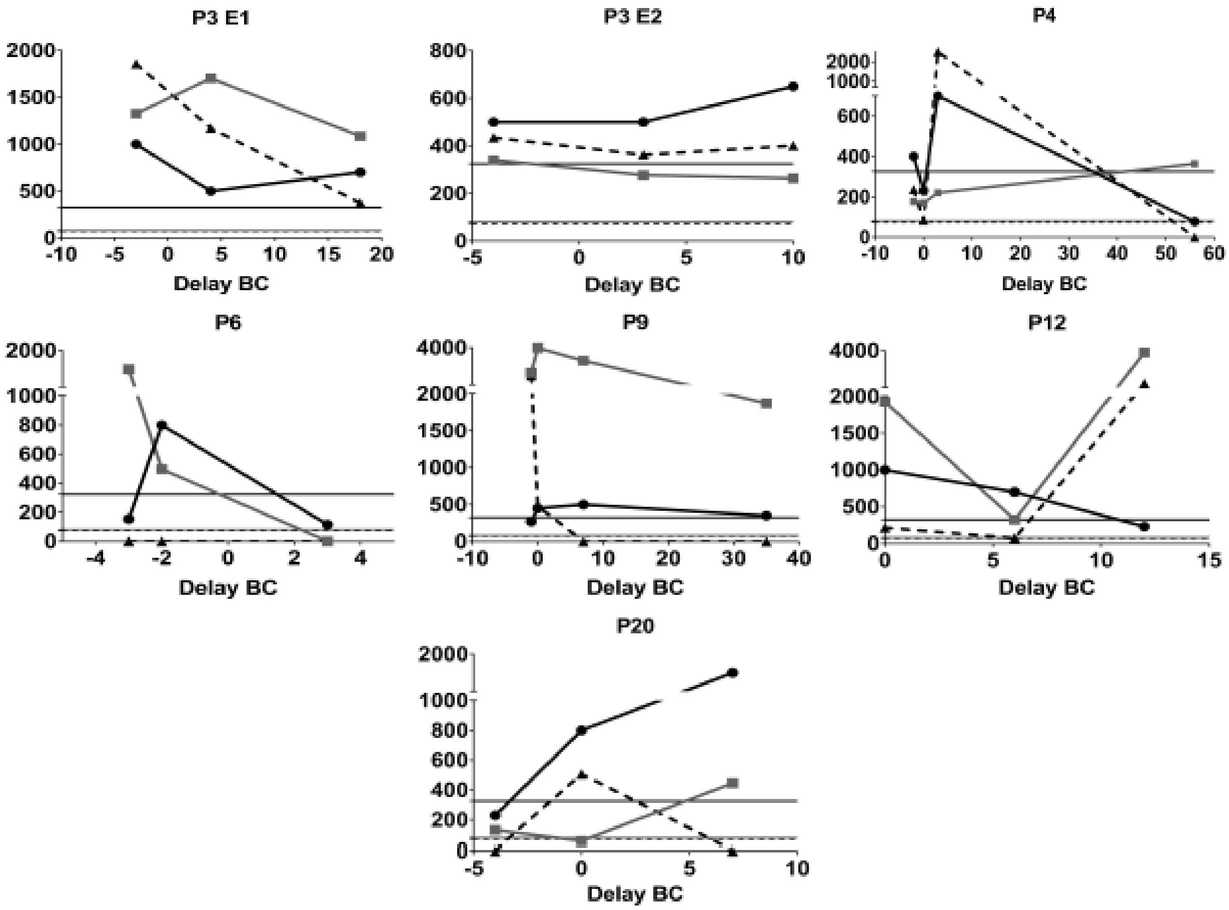

Kinetics of serum biomarkers: MS-DS index (black circles), Mnn (black triangles) and BDG (grey squares) in patients with IC. Day 0 indicates the date of *Candida* isolation from blood cultures. Mortality rate of IC patients was evaluated at 1-month and during the whole period of hospitalization. Horizontal lines indicated the cut-off values for BDG (grey line), Mnn (hatched black line) and MS-DS index (black line). Patients P6, P12 and P20 died within 1 month after positive blood cultures, patients P3, P9 died during their hospital stay and patient P4 survived. E1 and E2 correspond to the first and second candidaemia episodes in patient P3.

Figure S2.

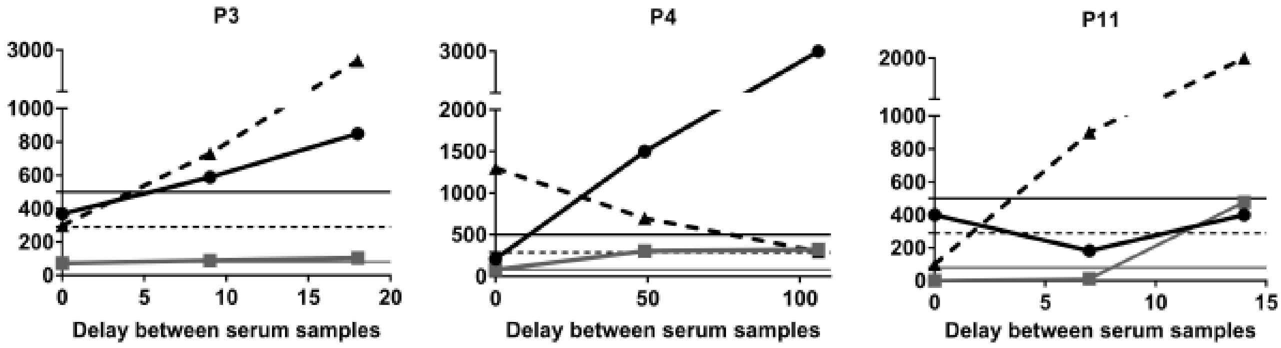

Kinetics of serum biomarkers: MS-DS index (black circles), GM (black triangles) and BDG (grey squares) in IA patients. Day 0 indicates the beginning of biomarker monitoring. Biomarkers levels are indicated on the left ordinate axis with reference to BDG values (raw values in pg/ML), MS-DS index and GM (index x 1000). Horizontal lines indicated the cut-off values for BDG (grey line), GM (hatched black line) and MS-DS index (black line).
